# Don’t Fool with Your Eyes

**Published:** 1884-12

**Authors:** 


### DON'T FOOL WITH YOUR EYES.

### *SUGGESTIONS FROM AN OPTICIAN ABOUT SELECTING EYEGLASSES AND SPECTACLES.*

"THERE is a great difference in the prices of eyeglasses and spectacles," an optician said. "You can buy a pair of eyeglasses for fifteen cents, while a pair that looks exactly like them will cost you $2. The difference between them is that by using one pair a man is very liable to ruin his sight, while the other pair will materially aid it."

"What makes the difference in value?"

"The quality of the glass and the amount of work used in polishing them. The cheap ones are generally made of very common glass, and are by no means perfect. Sometimes there are bubbles in them, and sometimes there are waving lines. You have looked through a window-glass that distorted everything, haven't you? Well, just imagine taking such kind of glass to improve your sight with. Persons are not half careful enough of their eyes, and the sight of thousands has been injured by using bad and unsuitable glasses. Poor quality glasses are injurious enough, but when a person looks through glasses that are both poor in quality and in no way fitted to improve his sight, he runs a terrible risk."

"Cannot one tell if the glasses help him?"

"It depends on circumstances. If a man finds that he is getting far sighted, and tries on a pair of far sighted glasses, and they make him see better at that time, he is apt to buy them. They may, however, be too old, and thus strain his sight, or they may be too young for him, and he does not receive the benefit that he should. By going to a first-class optician his sight would be tried in a proper manner, and he would get just the right kind of glasses. It is a very false economy to buy cheap glasses. I've seen a man stop at a stand in the street and buy a pair of glasses for a quarter, !ust by trying them on and looking at a newspaper. Lots of persons' eyes are not of the same strength, and ought to have glasses of different strength in the same frame. If a man with eyes of this character gets a ready-made pair of spectacles he is going to suffer."

---

You will never convince a man of ordinary sense by overbearing his understanding.

---

A vigorous mind is as necessarily accompanied with violent passions as a great fire with great heat.

